# ER–mitochondria associations are regulated by the VAPB–PTPIP51 interaction and are disrupted by ALS/FTD-associated TDP-43

**DOI:** 10.1038/ncomms4996

**Published:** 2014-06-03

**Authors:** Radu Stoica, Kurt J. De Vos, Sébastien Paillusson, Sarah Mueller, Rosa M. Sancho, Kwok-Fai Lau, Gema Vizcay-Barrena, Wen-Lang Lin, Ya-Fei Xu, Jada Lewis, Dennis W. Dickson, Leonard Petrucelli, Jacqueline C. Mitchell, Christopher E. Shaw, Christopher C. J. Miller

**Affiliations:** 1 Department of Neuroscience, Institute of Psychiatry, Kings College London, London SE5 8AF, UK; 2 Clinical Neurosciences, Institute of Psychiatry, Kings College London, London SE5 8AF, UK; 3 Centre for Ultrastructural Imaging, King’s College London, London SE5 8AF, UK; 4 Department of Neuroscience, Mayo Clinic, Jacksonville, Florida 32224, USA; 5 These authors contributed equally to this work; 6 Present address: Alzheimer’s Research UK, Cambridge CB21 6AD, UK; 7 Present address: Chinese University of Hong Kong, Shatin, NT, Hong Kong SAR; 8 Present address: Sheffield Institute for Translational Neuroscience, University of Sheffield, South Yorkshire S10 2HQ, UK

## Abstract

Mitochondria and the endoplasmic reticulum (ER) form tight structural associations and these facilitate a number of cellular functions. However, the mechanisms by which regions of the ER become tethered to mitochondria are not properly known. Understanding these mechanisms is not just important for comprehending fundamental physiological processes but also for understanding pathogenic processes in some disease states. In particular, disruption to ER–mitochondria associations is linked to some neurodegenerative diseases. Here we show that the ER-resident protein VAPB interacts with the mitochondrial protein tyrosine phosphatase-interacting protein-51 (PTPIP51) to regulate ER–mitochondria associations. Moreover, we demonstrate that TDP-43, a protein pathologically linked to amyotrophic lateral sclerosis and fronto-temporal dementia perturbs ER–mitochondria interactions and that this is associated with disruption to the VAPB–PTPIP51 interaction and cellular Ca^2+^ homeostasis. Finally, we show that overexpression of TDP-43 leads to activation of glycogen synthase kinase-3β (GSK-3β) and that GSK-3β regulates the VAPB–PTPIP51 interaction. Our results describe a new pathogenic mechanism for TDP-43.

## Abstract (web summary)

Mutations in the protein TDP-43 are implicated in amyotrophic lateral sclerosis. Here, the authors show that mutant TDP-43 perturbs endoplasmic reticulum (ER)–mitochondria associations by altering interactions between the mitochondrial protein PTPIP51 and the ER protein VAPB.

## Graphical abstract

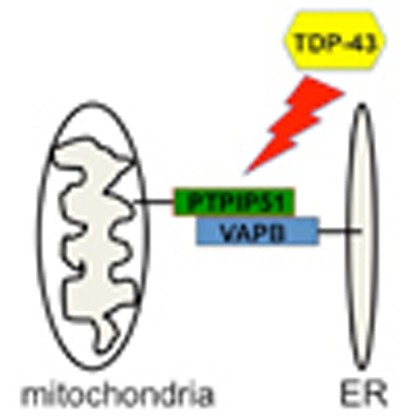

Mitochondria play pivotal roles in a number of cellular processes including energy metabolism, Ca^2+^ homeostasis and lipid metabolism. These functions require a dynamic spatial organization that permits relaying of signals to and from other organelles. In particular, mitochondria are associated with the endoplasmic reticulum (ER) with between 5 and 20% of the mitochondrial surface being closely apposed to ER membranes^1^,^2^. The regions of ER associated with mitochondria are termed mitochondria-associated ER membranes (MAMs) and these contacts facilitate a variety of signalling processes between the two organelles including Ca^2+^ and phospholipid exchange (see reviews refs 3, 4, 5). Indeed, ER–mitochondria associations are now believed to impact on a diverse number of physiological processes including ATP production, autophagy, protein folding in the ER, mitochondrial biogenesis and transport and apoptosis^4^,^5^,^6^,^7^,^8^.

Despite their fundamental importance to cell metabolism, the mechanisms that mediate ER–mitochondria associations are not properly understood. Electron microscopy (EM) studies reveal the presence of tethers that link ER and mitochondria^2^ but the biochemical makeup of these structures is not fully clear. In yeast, proteins of the ER–mitochondria encounter structure act as a molecular tether between ER and mitochondria, but orthologues in higher eukaryotes have not so far been identified^9^. There is evidence that mitofusin-2 links ER and mitochondria in mammalian cells^10^ but EM studies reveal that ER and mitochondria are adjoined by structures of differing length and this suggests that a number of proteins can physically connect these organelles^2^,^4^,^5^.

Recently, several studies have linked defective ER–mitochondria interactions to some neurodegenerative diseases^11^,^12^,^13^,^14^,^15^. Accumulations of TDP-43 are a common pathological feature in a variety of neurodegenerative diseases including amyotrophic lateral sclerosis (ALS) and fronto-temporal dementia (FTD)^16^. The importance of TDP-43 in the disease process is highlighted by the findings that mutations in the *TDP-43* gene are causative for some familial forms of ALS/FTD^17^. However, the mechanisms by which TDP-43 contributes to the neurodegenerative process are not properly understood and indeed, disruption to a variety of physiological pathways have been implicated in the pathogenic process (see reviews refs 18, 19).

Recently, we identified the outer mitochondrial membrane protein, protein tyrosine phosphatase-interacting protein-51 (PTPIP51) as a binding partner for the resident ER protein vesicle-associated membrane protein-associated protein-B (VAPB)^20^. Here, we show that the VAPB–PTPIP51 interaction regulates ER–mitochondria associations. Moreover, we demonstrate that expression of both wild-type and ALS/FTD-associated mutant TDP-43 perturbs ER–mitochondria associations and that this is accompanied by changes to the VAPB–PTPIP51 interaction. As such, our findings reveal a new mechanism for controlling ER–mitochondria interactions and highlight ER–mitochondria associations as a target for disruption by TDP-43.

## Results

### VAPB and PTPIP51 interact in a range of biochemical assays

**Figure 1:**
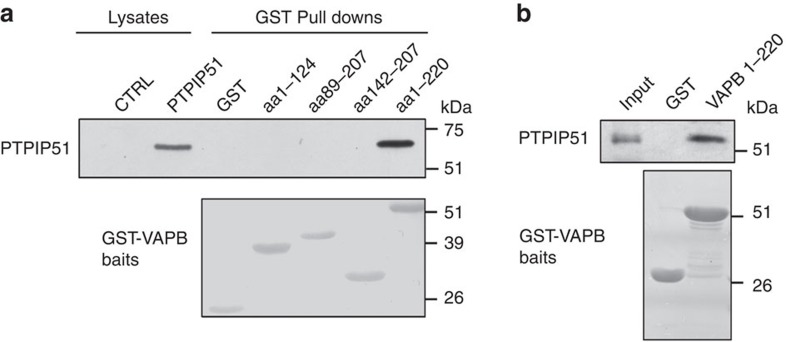
Binding of VAPB to PTPIP51 requires its complete cytosolic domain. (**a**) Cells were transfected with either control empty vector (CTRL) or HA-tagged PTPIP51 and the lysates then used in GST pull-down assays with either GST, GST-VAPB1-124 (the MSP domain), GST-VAPB89-207, GST-VAPB142-207 (containing the coiled-coil domain) or GST-VAPB1-220 (the entire cytosolic domain) ‘baits’. PTPIP51 only bound GST-VAPB1-220. Upper shows immunoblot of both lysates and GST pulldowns probed for PTPIP51 via the HA tag; lower shows Poncea red-stained blot of GST ‘baits’. (**b**) VAPB and PTPIP51 cytosolic domains bind directly *in vitro*. PTPIP51 cytosolic domain (amino acids 36–470) produced in *E. coli* was incubated with either GST or GST-VAPB1-220 (the entire cytosolic domain) and bound PTPIP51 detected by immunoblotting. Upper shows input and either GST or GST-VAPB1-220 pulldown; lower shows Coomassie blue-stained gel of GST ‘baits’.

VAPB and PTPIP51 interact in a variety of biochemical assays, and the region of PTPIP51 that mediates binding involves its central coiled-coil domain (amino acids 84–174)^20^. To gain insight into the domain(s) of VAPB involved in binding PTPIP51, we generated glutathione *S*-transferase (GST) fusion proteins comprising the entire VAPB cytoplasmic domain (amino acids 1–220), its *N*-terminal major sperm protein domain (amino acids 1–124), its central coiled-coil domain (amino acids 142–207) and sequences encompassing the C-terminal part of VAPB (amino acids 89–207) and tested their abilities to bind to PTPIP51 in pull-down assays from PTPIP51-transfected cells. Only the entire VAPB cytoplasmic domain bound PTPIP51 (Fig. 1a). Hence, the entire cytoplasmic domain of VAPB is required for binding to PTPIP51 or the interaction requires some topographical conformation that is lost in the smaller VAPB fragments. We also performed *in vitro*-binding studies with purified VAPB and PTPIP51 cytoplasmic domains that were generated in *E. coli*. PTPIP51 cytoplasmic domain (amino acids 36–470) was incubated with either GST or GST-VAPB and the GST moiety used to isolate bound PTPIP51. GST-VAPB cytoplasmic domain but not GST bound to PTPIP51 in these assays (Fig. 1b). Together with our previous studies^20^, these results demonstrate that VAPB interacts with PTPIP51 in a number of different assays including *in vitro* assays in the absence of other proteins.

### VAPB and PTPIP51 mediate ER–mitochondria associations

The binding of VAPB to PTPIP51 suggests that these proteins act as tethers to link ER with mitochondria. We therefore monitored how modulating VAPB and PTPIP51 expression affects ER–mitochondria associations via EM in mouse NSC34 motor neuron cells. We quantified ER–mitochondria associations by determining the proportion of the mitochondrial surface that was closely apposed (<30 nm) to ER. Such an approach has been used by others^21^,^22^.

**Figure 2:**
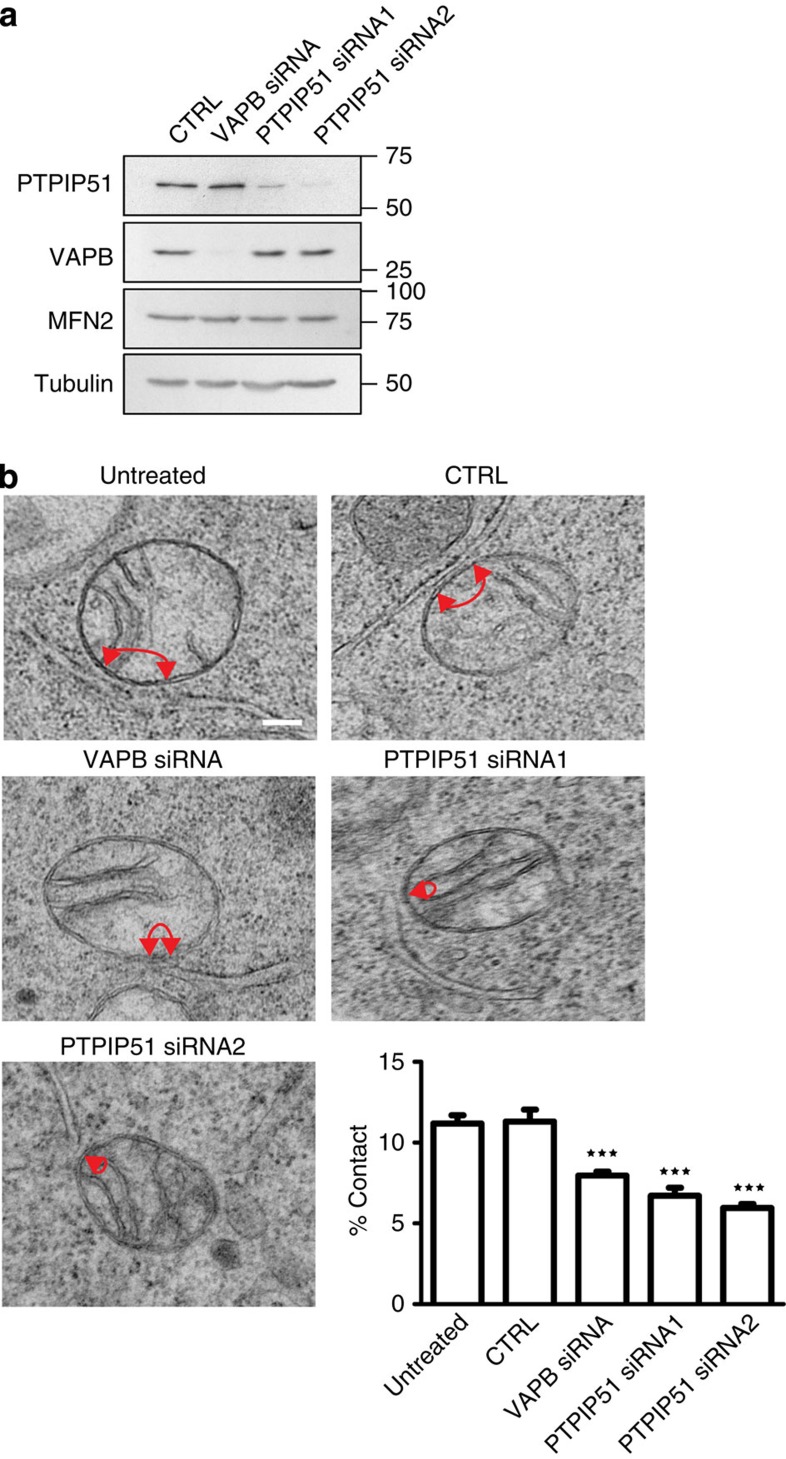
siRNA knockdown of VAPB or PTPIP51 reduces ER–mitochondria associations in NSC34 cells. (**a**) Immunoblots showing siRNA knockdown of VAPB and PTPIP51. siRNA knockdown of VAPB does not affect expression of PTPIP51, siRNA knockdown of PTPIP51 does not affect expression of VAPB and siRNA knockdown of either VAPB or PTPIP51 does not affect expression of mitofusin-2 (MFN2). Also shown is an immunoblot of tubulin as a loading control. (**b**) Representative electron micrographs of ER–mitochondria associations in untreated cells and control (CTRL), VAPB or PTPIP51 siRNA-treated cells; arrowheads with loops show regions of association. Scale bar, 100 nm. Bar chart shows % of the mitochondrial surface closely apposed to ER in the different samples. Data were analysed by one-way analysis of variance followed by Tukey’s multiple comparison test. *N*=27–35 cells and 279–374 mitochondria; error bars are s.e.m.; ***P<0.001.

We first reduced VAPB and PTPIP51 expression using short interfering RNAs (siRNAs) and identified one potent mouse VAPB siRNA and two PTPIP51 siRNAs. Consistent with previous studies in HEK293 cells^20^, siRNA knockdown of VAPB did not influence expression of PTPIP51 and knockdown of PTPIP51 did not influence expression of VAPB in NSC34 cells (Fig. 2a). Approximately 12% of the mitochondrial surface was closely associated with ER in untreated and control siRNA cells. However, siRNA knockdown of either VAPB or PTPIP51 significantly reduced these values (Fig. 2b). We also monitored how loss of VAPB and PTPIP51 affected ER–mitochondria associations in human HEK293 cells using previously characterized siRNAs that involved different sequences^20^. Again, depletion of VAPB and PTPIP51 reduced ER–mitochondria associations (% mitochondrial surface closely apposed to ER reduced by 29% in VAPB siRNA and 46% in PTPIP51 siRNA-treated cells compared with control siRNA cells. Control vs VAPB siRNA *P*<0.05; control vs PTPIP51 siRNA *P*<0.001. Data analysed by one-way analysis of variance and Tukey’s multiple comparison test. *N*=22 cells for each treatment and 245–352 mitochondria).

**Figure 3:**
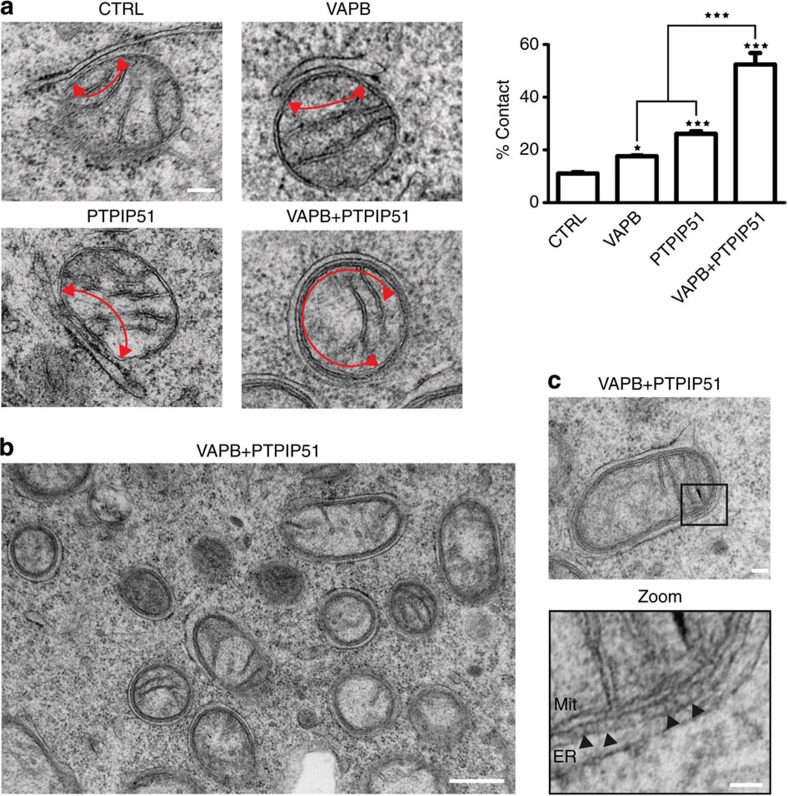
Overexpression of VAPB and/or PTPIP51 increases ER–mitochondria associations in NSC34 cells. (**a**) Representative electron micrographs of ER–mitochondria associations in cells transfected with control ECFP vector (CTRL), ECFP-VAPB (VAPB), PTPIP51 (PTPIP51) or ECFP-VAPB+PTPIP51; arrowheads with loops show regions of association. (**b**) Low-magnification electron micrograph of cells co-transfected with VAPB and PTPIP51 to show numerous mitochondria with closely apposed ER. (**c**) With a zoom view; ER–mitochondria contacts in VAPB+PTPIP51 co-transfected cells showing tight associations of ER and mitochondria (Mit) as indicated with putative connections (arrowheads) linking ER with the outer mitochondrial membrane. Bar chart in **a** shows % of the mitochondrial surface closely apposed to ER in the different samples. Data were analysed by one-way analysis of variance followed by Tukey’s multiple comparison test. *N*=30–32 cells and 376–483 mitochondria; error bars are s.e.m.; *P<0.05, ***P<0.001. Scale bars, 100 nm in (**a**) 500 nm in (**b**) and 30 nm in (**c**) zoom.

We next elevated the expression of VAPB and PTPIP51 via transfection. Cells were transfected with either enhanced cyan fluorescent protein (ECFP) control vector, ECFP-VAPB or PTPIP51 that was expressed using a bicistronic internal ribosome entry site vector with AcGFP. The ECFP/AcGFP tags were then utilized to isolate transfected cells using a cell sorter. The proportion of the mitochondrial surface closely associated with ER in control cells expressing ECFP alone was not significantly different to that in untreated cells or cells treated with control siRNA (Fig. 3a). However, expression of VAPB or PTPIP51 markedly increased ER–mitochondria associations (Fig. 3a). We also monitored the effect of co-transfecting both VAPB and PTPIP51 and obtained a remarkable phenotype where large proportions of the mitochondrial surface were closely associated with ER (Fig. 3a,b). Analyses of these images revealed the presence of structures that appeared to tether ER with mitochondria (Fig. 3c); the average spacing between ER and mitochondria in the VAPB+PTPIP51 co-transfected cells was 14.7 nm.

**Figure 4:**
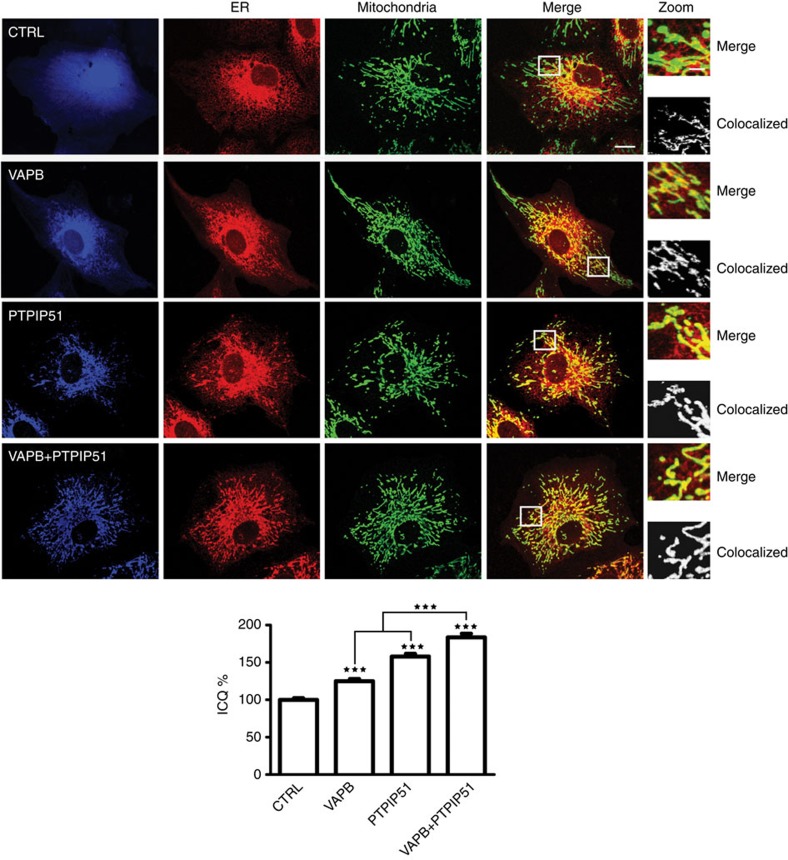
Overexpression of VAPB and/or PTPIP51 increases ER–mitochondria associations in CV-1 cells. Confocal immunostaining of CV-1 cells transfected with control ECFP vector (CTRL; blue), ECFP-VAPB (VAPB; blue), PTPIP51-HA (PTPIP51; blue) or ECFP-VAPB+PTPIP51-HA (VAPB+PTPIP51) and immunostained for ER (using mouse PDI antibody) and mitochondria (using rabbit TOM-20) as indicated. In VAPB+PTPIP51 co-transfected cells, only ECFP-VAPB (blue) labelling is shown but duplicate coverslips were immunostained for ECFP-VAPB and PTPIP51-HA, and this demonstrated that ~95% of cells expressed both transfected proteins. These data are consistent with many previous reports, which show that most co-transfected cells express both plasmids (for example, ref. 59) Merge is of ER and mitochondria labelling only; the zoom view shows co-localized pixels. Scale bar, 20 μm. Bar chart shows intensity correlation quotient (ICQ) values (with ECFP control represented as 100%) in the different transfections. Data were analysed by one-way analysis of variance with Tukey’s *post hoc* test. *N*=34–52 cells; error bars are s.e.m.; ***P<0.001.

Confocal microscopy has also been utilized to study ER–mitochondria interactions (for example, refs 1, 10). Since overexpressing VAPB and PTPIP51 produced particularly striking EM images, we reasoned that such phenotypes would be clearly discernible by light microscopy. We therefore monitored the effect of transfecting VAPB and/or PTPIP51 on ER mitochondria co-localization by confocal microscopy. For these investigations, we utilized CV1 cells since these have a flattened and spread morphology, which facilitates such morphological analyses. Cells were transfected with ECFP control, ECFP-VAPB, haemagglutin-tagged PTPIP51 (PTPIP51-HA) or ECFP-VAPB+PTPIP51-HA, and ER and mitochondria co-localization monitored by immunostaining. ER was detected by labelling for protein disulphide isomerase (PDI) and mitochondria by labelling for translocase of the outer mitochondrial membrane protein-20 (TOM20). Transfection of VAPB and/or PTPIP51 all noticeably increased co-localization of ER and mitochondria and in agreement with the EM studies, this phenotype was particularly striking in VAPB+PTPIP51 co-transfected cells (Fig. 4). To obtain unbiased quantitative data on the effects of VAPB and PTPIP51 overexpression on ER–mitochondria co-localization, we used intensity correlation analysis (ICA) to analyse the co-localization of PDI and TOM20 signals in the different samples^23^. ICA is one of the most rigorous methods for studying co-localization of proteins by confocal microscopy since it compares the scatter plots of two stains against the product of the difference of the pixel intensities of each of the two stains from their respective means. Thus, unlike other methods, which simply compare co-localization of pixels, ICA also determines whether the pixel intensities from the two signals vary in synchrony. ICA yields an intensity correlation quotient, which is a statistically testable single-value assessment of the relationship between two stained protein pairs^23^. Transfection of VAPB, PTPIP51 and VAPB+PTPIP51 all significantly increased co-localization of ER and mitochondria in these assays (Fig. 4). Thus, modulating VAPB and PTPIP51 expression significantly influences ER–mitochondria interactions in both EM and confocal light microscopy assays.

### TDP-43 disrupts ER–mitochondria associations

**Figure 5:**
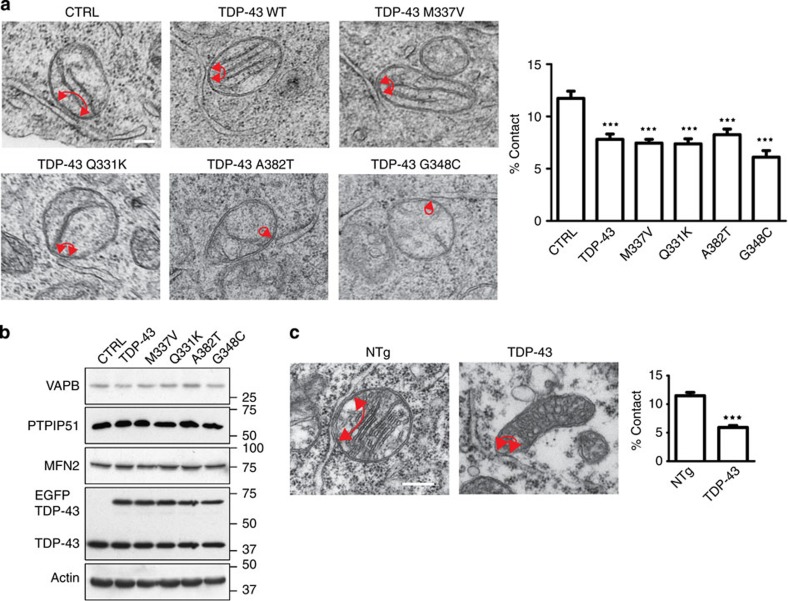
Expression of TDP-43 reduces ER–mitochondria associations. (**a**) Representative electron micrographs of ER–mitochondria associations in NSC34 cells transfected with control EGFP vector (CTRL), EGFP-TDP-43, EGFP-TDP-43M337V, EGFP-TDP-43Q331K, EGFP-TDP-43A382T or EGFP-TDP-43G348C as indicated; arrowheads with loops show regions of association. Scale bar, 100 nm. Bar chart shows % of the mitochondrial surface closely apposed to ER in the different samples. Data were analysed by one-way analysis of variance followed by Tukey’s multiple comparison test. *N*=30–35 cells and 352–481 mitochondria; error bars are s.e.m.; ***P<0.001. (**b**) Expression of wild-type or disease-associated mutant TDP-43 does not alter expression of VAPB, PTPIP51 or mitofusin-2 (MFN2). Immunoblots of NSC34 cells transfected with EGFP as a control (CTRL), or wild-type or mutant EGFP-TDP-43 as indicated. Transfected cells were purified via EGFP using a cell sorter and the samples probed on immunoblots as indicated. On TDP-43 immunoblot, upper species is transfected and lower species endogenous TDP-43; actin is shown as a loading control. (**c**) Representative electron micrographs of ER–mitochondria associations in motor neurons of TDP-43 transgenic mice and their non-transgenic littermates; arrowheads with loops show regions of association. Scale bar, 200 nm. Bar chart shows % of the mitochondrial surface closely apposed to ER in the two samples. Data were analysed by the unpaired *t*-test. *N*=67–88 cells and 438–749 mitochondria; error bars are s.e.m.; ***P<0.001.

**Figure 6:**
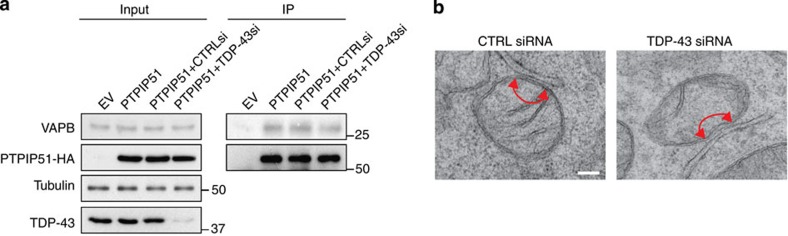
siRNA loss of TDP-43 does not affect binding of PTPIP51 to VAPB or ER–mitochondria associations in NSC34 cells. (**a**) Loss of TDP-43 does not affect the binding of PTPIP51 to VAPB. Cells were transfected with empty vector (EV), PTPIP51-HA or PTPIP51-HA+either control (CTRL) or TDP-43 siRNAs (mixture of two siRNAs). PTPIP51 was immunoprecipitated using the HA tag and the amounts of endogenous bound VAPB detected by immunoblotting. No signals were obtained for either VAPB or PTPIP51 in immunoprecipitations from EV-transfected cells, which demonstrates the specificity of the immunoprecipitations. Both inputs and immunoprecipitations (IP) are shown. Data analysed by one-way analysis of variance; *N*=3. (**b**) Representative electron micrographs of ER–mitochondria associations in control (CTRL) and TDP-43 siRNA-treated cells. Arrowheads with loops show regions of association. Scale bar, 100 nm. Data were analysed by the unpaired *t*-test. *N*=31 cells each for both control and TDP-43 siRNAs, and 252 and 283 mitochondria.

We next enquired whether expression of ALS/FTD-associated TDP-43 influenced ER–mitochondria associations. To do so, we transfected NSC34 cells with EGFP control vector, EGFP-TDP-43, or familial ALS mutant EGFP-TDP-43M337V, TDP-43Q331K, TDP-43A382T or TDP-43G348C. Transfected cells were again isolated using a cell sorter via the EGFP tags and ER–mitochondria associations were quantified by EM as described above. We detected no change in the numbers of mitochondria or ER profiles in the presence of either wild-type or mutant TDP-43. However, expression of both wild-type and mutant TDP-43 all led to significant reductions in ER–mitochondria associations (Fig. 5a). The finding that both wild-type TDP-43 and mutant TDP-43 disrupted ER–mitochondria interactions is consistent with the phenotypes seen in transgenic mice, where expression of both wild-type and mutant TDP-43 all induce disease^24^,^25^,^26^,^27^,^28^. We also enquired whether siRNA-mediated loss of TDP-43 influenced ER–mitochondria associations or the VAPB–PTPIP51 interaction. However, loss of TDP-43 had no effect on either of these (Fig. 6).

We next monitored whether transfection of wild-type or mutant TDP-43 influenced expression of VAPB, PTPIP51 or mitofusin-2, a further protein that has been linked to ER–mitochondria associations^10^. To do so, cell-sorted control EGFP and EGFP-TDP-43-transfected cell proteins were probed on immunoblots for VAPB, PTPIP51 and mitofusin-2. However, no differences in expression of any of these proteins were detected in the different samples (Fig. 5b). Thus, the reduction in ER–mitochondria interactions seen in cells expressing wild-type TDP-43 or mutant TDP-43 is not due to changes in expression of VAPB, PTPIP51 or mitofusin-2.

To complement the above studies, we also quantified ER–mitochondria associations in spinal cord motor neurons of 1-month-old homozygous TDP-43 transgenic and their non-transgenic littermates^26^. At this age, these TDP-43 transgenic mice develop motor defects and symptoms characteristic of ALS^26^. Similar to control NSC34 cells, ~12% of the mitochondrial surface was closely apposed to ER in the non-transgenic animals. However, in TDP-43 transgenic mice, ER–mitochondria interactions were significantly reduced (Fig. 5c).

### TDP-43 disrupts VAPB–PTPIP51 interactions

**Figure 7:**
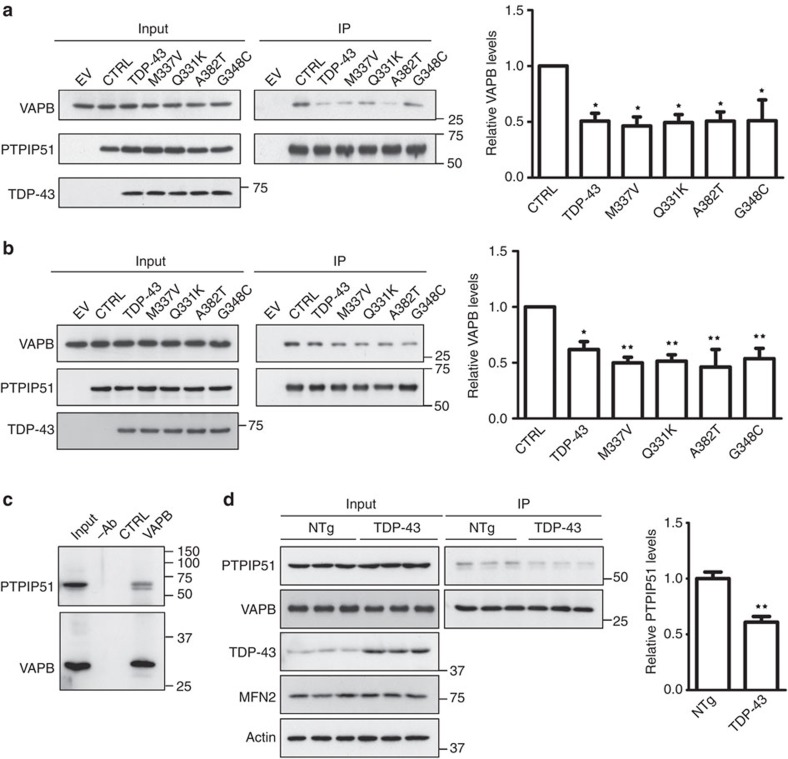
Expression of TDP-43 reduces the binding of VAPB to PTPIP51 in both transfected cells and transgenic mice. (**a**,**b**) Cells were transfected with either empty vector (EV) or co-transfected with PTPIP51-HA and either EGFP control vector (CTRL) or wild-type or ALS/FTD mutants of TDP-43 (TDP-43M337V, TDP-43Q331K, TDP-43A382T and TDP-43G348C). PTPIP51 was immunoprecipitated using the HA tag and the amounts of endogenous bound VAPB detected by immunoblotting. No signals were obtained for either VAPB or PTPIP51 in immunoprecipitations from EV-transfected cells, which demonstrates the specificity of the immunoprecipitations. Both inputs and immunoprecipitations (IP) are shown. The bar chart shows relative levels of VAPB bound to PTPIP51 in the immunoprecipitations following quantification of signals from immunoblots. VAPB signals were normalized to immunoprecipitated PTPIP51-HA signals. (**a**) Shows data for NSC34 cells; *N*=3. (**b**) Shows data for HEK293 cells; *N*=5. Data were analysed by one-way analysis of variance and Tukey’s *post hoc* test; error bars are s.e.m., *P<0.05, **P<0.01. (**c**) VAPB binds to PTPIP51 in mouse spinal cords. VAPB was immunoprecipitated using rabbit anti-VAPB and detected on immunoblots using rat anti-VAPB; bound PTPIP51 was detected using rat PTPIP51. Both inputs and immunoprecipitations (IP) are shown. Control immunoprecipitations were performed using no primary antibody (−Ab) or preimmune rabbit serum (CTRL). (**d**) Reduced binding of VAPB to PTPIP51 in TDP-43 transgenic mice. Immunoprecipitations were performed as in **c** from three non-transgenic (NTg) and three TDP-43 transgenic mice. Also shown are immunoblots for TDP-43, mitofusin-2 (MFN2) and actin as a loading control in the spinal cords. Bar chart shows relative levels of PTPIP51 bound to VAPB in the immunoprecipitations following quantification of signals from immunoblots. PTPIP51 signals were normalized to immunoprecipitated VAPB signals. Data were analysed by the unpaired *t*-test; error bars are s.e.m., **P<0.01.

Since VAPB and PTPIP51 mediate ER–mitochondria associations, and since expression of TDP-43 disrupted ER–mitochondria associations, we enquired whether the expression of TDP-43 influenced binding of VAPB to PTIP51. To do so, we transfected NSC34 cells with PTPIP51-HA and either EGFP control vector or wild-type or ALS mutants of EGFP-TDP-43, and monitored the amounts of endogenous VAPB bound to PTPIP51-HA in immunoprecipitation assays. Consistent with the EM studies, expression of both wild-type and ALS mutant TDP-43 all reduced the amount of VAPB that was bound to PTPIP51 (Fig. 7a). We obtained similar results in HEK293 cells (Fig. 7b).

We also monitored the effect of TDP-43 on the VAPB–PTPIP51 interaction in TDP-43 transgenic mice using immunoprecipitation assays. In spinal cords, endogenous VAPB bound to endogenous PTPIP51 in these assays (Fig. 7c), which is consistent with previous results on endogenous VAPB–PTPIP51 binding in other tissues^20^. In line with the data from the TDP-43-transfected NSC34 cells, no differences in the levels of VAPB, PTPIP51 or mitofusin-2 were detected in spinal cords of TDP-43 and non-transgenic mice (Fig. 7d). However, the amounts of PTPIP51 bound to VAPB were significantly reduced in the TDP-43 transgenic mice (Fig. 7d). Thus, expression of ALS/FTD-associated TDP-43 disrupts ER–mitochondria interactions and this is associated with a decrease in the binding of VAPB to PTPIP51.

### Overexpression of TDP-43 disturbs cellular Ca^2+^ homeostasis

**Figure 8:**
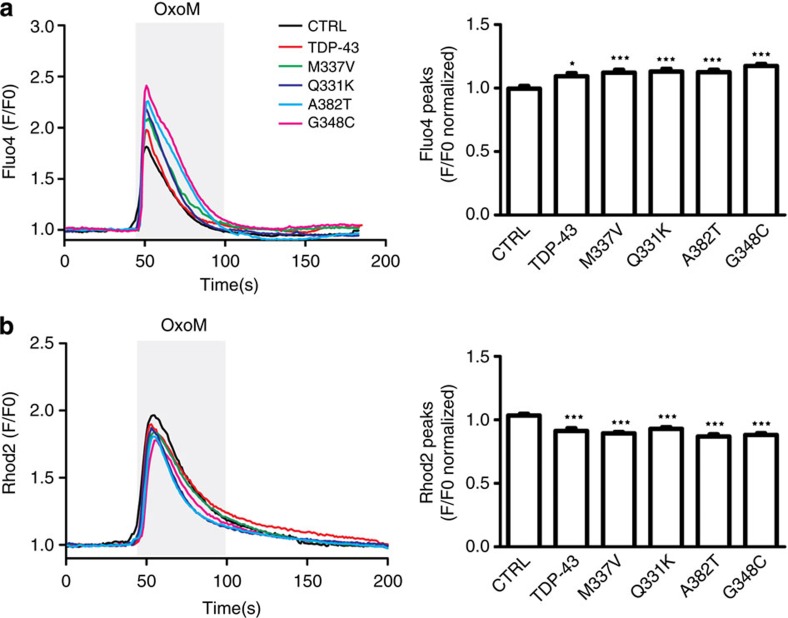
Expression of TDP-43 disrupts cellular Ca^2+^ homeostasis. HEK293 cells were transfected with M3R and either control empty vector (CTRL), TDP-43, TDP-43M337V, TDP-43Q331K, TDP-43A382T or TDP-43G348C as indicated. Release of ER Ca^2+^ was induced by treatment of cells with Oxotremorine-M (OxoM). (**a**) shows cytosolic Ca^2+^ levels with representative Fluo4 fluorescence traces on the left and normalized peak values on the right. Fluo4 fluorescence shows a transient increase in cytosolic Ca^2+^ levels upon OxoM treatment but compared with control, wild-type and mutant TDP-43 all increase peak cytosolic Ca^2+^ levels. (**b**) shows mitochondrial Ca^2+^ levels with representative Rhod2 fluorescence traces on the left and normalized peak values on the right. Data were analysed by one-way analysis of variance and Tukey’s *post hoc* test. (**a**) *N*=48–52 cells from 3–5 experiments; (**b**) *N*=48–52 cells from 3–4 experiments. *P<0.05, ***P<0.001; error bars are s.e.m.

Both ER and mitochondria are stores for intracellular Ca^2+^ and the ER–mitochondria interface, and MAMs regulate Ca^2+^ exchange between the two organelles^1^,^2^,^10^,^20^. Indeed, we have shown that siRNA loss of VAPB and PTPIP51 perturbs cellular Ca^2+^ homeostasis following its release from ER stores^20^. The TDP-43-induced disruption to the VAPB–PTPIP51 interaction and ER–mitochondria associations thus predict that TDP-43 may influence Ca^2+^ homeostasis. We therefore monitored the effect of overexpressing TDP-43 on cytosolic and mitochondrial Ca^2+^ levels after induction of inositol 1,4,5-trisphosphate receptor (IP3R)-mediated Ca^2+^ release from ER stores. For these experiments, we used HEK293 cells co-transfected with the M3 muscarinic-ACh-receptor (M3R) and either control vector or TDP-43, and triggered physiological IP3R-mediated Ca^2+^ release from ER stores by application of the M3R agonist oxotremorine-M. In line with previous studies on VAPB, PTPIP51 and ER–mitochondria associations, we used HEK293 cells for these experiments since they do not express endogenous M3R and so provide a useful model for monitoring cytosolic and mitochondrial Ca^2+^ levels after release from ER^20^. Transfection of both wild-type and mutant TDP-43 all induced significant increases in cytosolic Ca^2+^ and decreases in mitochondrial Ca^2+^ levels (Fig. 8). Such findings are consistent with the observed TDP-43-induced decreases in ER–mitochondria and VAPB–PTPIP51 interactions^20^.

### TDP-43 activates GSK-3β to inhibit VAPB–PTPIP51 binding

**Figure 9:**
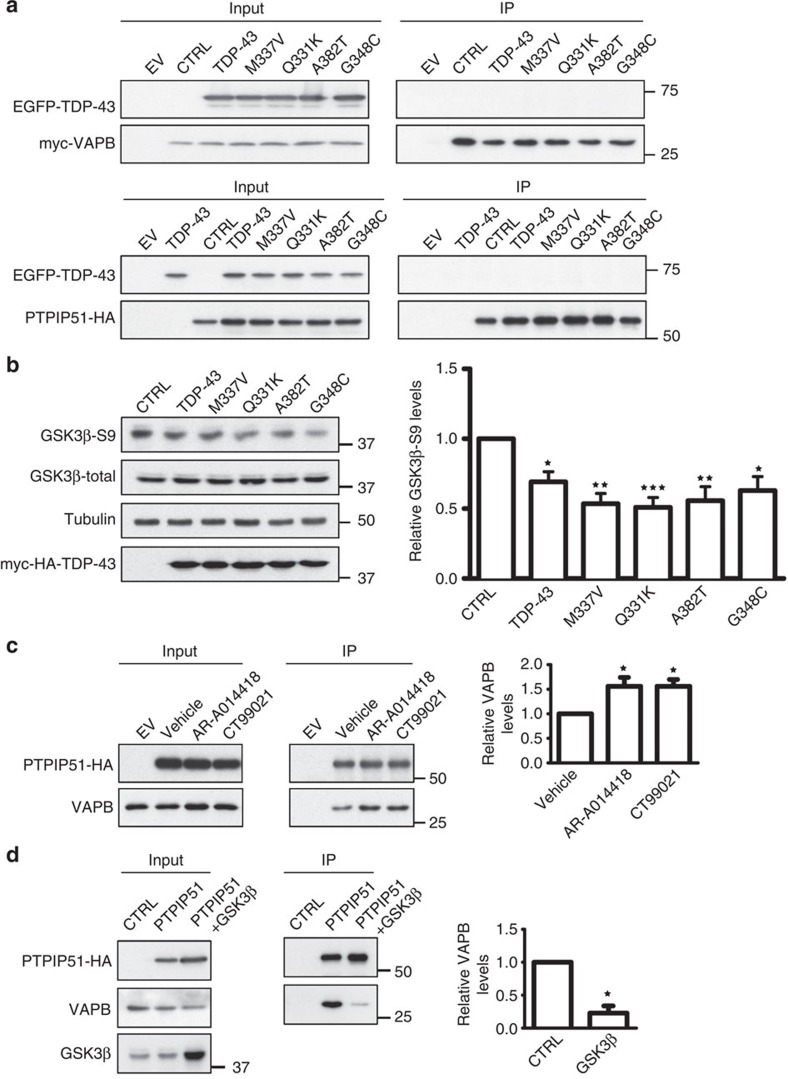
TDP-43 activates GSK-3β, and modulating GSK-3β activity regulates the VAPB–PTPIP51 interaction. (**a**) Immunoprecipitation assays of TDP-43 with VAPB and PTPIP51. HEK293 cells were transfected with either VAPB (upper) or PTPIP51 (lower) and either control empty vector (EV) or EGFP-tagged TDP-43, TDP-43M337V, TDP-43Q331K, TDP-43A382T or TDP-43G348C. VAPB and PTPIP51 were immunoprecipitated via their myc or HA tags and the samples probed for co-immunoprecipitating TDP-43 on immunoblots. (**b**) TDP-43 activates GSK-3β. HEK293 cells were transfected with either control empty vector (CTRL), TDP-43, TDP-43M337V, TDP-43Q331K, TDP-43A382T or TDP-43G348C and the samples probed on immunoblots for total GSK-3β and GSK-3β phosphorylated on serine-9. Phosphorylation of GSK-3β serine-9 is the principal mechanism for regulating its activity; serine-9 phosphorylation inhibits GSK-3β activity. Bar chart shows relative levels of GSK-3β serine-9 phosphorylation following quantification of signals from immunoblots and normalization to total GSK-3β signals. Data were analysed by one-way analysis of variance (ANOVA) and Tukey’s *post hoc* test. *N*=5; *P<0.05, **P<0.01,***P<0.001; error bars are s.e.m.. (**c**) Inhibition of GSK-3β increases the amount of VAPB bound to PTPIP51. Cells were transfected with control empty vector (EV) or HA-tagged PTPIP51 and treated with either vehicle, GSK-3β inhibitor AR-A014418 (1 μM) or GSK-3β inhibitor CT99021 (100 nM) for 16 h. PTPIP51 was immunoprecipitated using the HA tag and the amounts of endogenous bound VAPB detected by immunoblotting. Both inputs and immunoprecipitations (IP) are shown. Bar chart shows relative levels of VAPB bound to PTPIP51 in the immunoprecipitations following quantification of signals from immunoblots. VAPB signals were normalized to immunoprecipitated PTPIP51-HA signals. Data were analysed by one-way ANOVA and Tukey’s *post hoc* test; *N*=3, error bars are s.e.m., *P<0.05. (**d**) Transfection of GSK-3β decreases the amount of VAPB bound to PTPIP51. Cells were transfected with empty vector (EV), HA-PTPIP51 or HA-PTPIP51+GSK-3β. PTPIP51 was immunoprecipitated using the HA tag and the amounts of endogenous bound VAPB detected by immunoblotting. Both inputs and immunoprecipitations (IP) are shown. Bar chart shows relative levels of VAPB bound to PTPIP51 in the immunoprecipitations following quantification of signals from immunoblots. VAPB signals were normalized to immunoprecipitated PTPIP51 signals. Data were analysed by the unpaired *t*-test; *N*=3, error bars are s.e.m., *P<0.05.

To gain insight into the mechanism by which TDP-43 might influence the VAPB–PTPIP51 interaction, we first tested whether TDP-43 bound to either VAPB or PTPIP51. To do so, we performed immunoprecipitation assays from TDP-43+VAPB and TDP-43+PTPIP51-transfected cells. However, we did not detect binding of TDP-43 to either protein in these assays (Fig. 9a) or in yeast 2-hybrid library screens with VAPB or PTPIP51 baits^20^ (and our unpublished data). Others have also reported that TDP-43 does not bind VAPB^29^.

Phosphorylation is a common mechanism for controlling protein–protein interactions, and TDP-43 has recently been shown to activate glycogen synthase kinase-3β (GSK-3β)^30^. A major route for regulating GSK-3β is by inhibitory phosphorylation of serine-9, and so we monitored GSK-3β serine-9 phosphorylation in control and TDP-43-transfected cells by immunoblotting. In agreement with previous studies^30^, we found that overexpression of wild-type and four different TDP-43 mutants all activated GSK-3β as evidenced by reduced serine-9 phosphorylation (Fig. 9b). To determine whether GSK-3β might regulate the VAPB–PTPIP51 interaction, we modulated GSK-3β activity in transfected cells and monitored the effects on binding of VAPB to PTPI51 again using immunoprecipitation assays. Two structurally different GSK-3β inhibitors both increased, whereas transfection of GSK-3β to elevate its activity^31^ reduced binding of VAPB to PTPIP51 (Fig. 9c,d). Thus, TDP-43 activates GSK-3β and GSK-3β regulates the VAPB–PTPIP51 interaction.

## Discussion

Despite their fundamental importance to a variety of physiological processes, the mechanisms that mediate and regulate ER–mitochondria associations are not properly understood. Recently, we demonstrated an interaction between the resident ER protein VAPB and the mitochondrial protein PTPIP51 (ref. 20). VAPB is anchored in the ER via a C-terminal membrane-spanning domain such that its *N* terminus projects into the cytoplasm, and PTPIP51 is an outer mitochondrial membrane protein whose *C* terminus projects into the cytoplasm^20^. VAPB is also a MAM protein^20^. VAPB and PTPIP51 interact in yeast 2-hybrid, immunoprecipitation (including transfected and endogenous proteins), GST pull-down assays and proximity ligation assays, and recombinant VAPB and PTPIP51 cytoplasmic domains interact *in vitro* in the absence of other proteins demonstrating direct binding^20^ (and results presented here). Thus, VAPB and PTPIP51 represent plausible candidates for mediating ER–mitochondria interactions.

To directly test this possibility, we used EM to monitor how modulating VAPB and PTPIP51 expression affects ER–mitochondria associations. siRNA knockdown of VAPB or PTPIP51 reduced, whereas overexpression of VAPB and/or PTPIP51 markedly increased ER–mitochondria associations. Indeed, overexpression of both VAPB and PTPIP51 produced a remarkable phenotype detectable by both EM and confocal microscopy, in which ER and mitochondria redistribute to form close associations. Alterations to ER–mitochondria interactions influence Ca^2+^ exchange between these organelles, and we have demonstrated previously that modulating VAPB and PTPIP51 expression induces such changes to Ca^2+^ homeostasis^20^. Together, these findings support the conclusion that VAPB and PTPIP51 form at least one of the scaffolds that mediate ER–mitochondria associations.

Abnormal TDP-43 metabolism is strongly linked to both ALS and FTD. Thus, mutations in TDP-43 cause some familial forms of ALS and FTD, accumulations of TDP-43 are a hallmark pathology of ALS/FTD and overexpression of both wild-type and familial ALS-associated mutants of TDP-43 all induce disease in transgenic rodents^17^,^24^,^25^,^26^,^27^,^28^,^32^. Despite these findings, the mechanisms underlying TDP-43 toxicity are not properly known. Although TDP-43 is a predominantly nuclear protein, a proportion is normally present in the cytoplasm^33^,^34^,^35^. Cytoplasmic TDP-43 is associated with mitochondria but there is also evidence linking it with the ER^35^,^36^,^37^,^38^,^39^. Indeed, several recent studies have linked TDP-43 toxicity with damage to mitochondria^26^,^28^,^35^,^40^ and ER stress signalling pathways^36^,^37^,^38^,^39^. Together, these findings suggest that the ER–mitochondria axis may be perturbed by TDP-43.

In support of this notion, we show here that overexpression of wild-type and mutant TDP-43 reduce ER–mitochondria interactions, and that these reductions are associated with decreased binding of VAPB to PTPIP51. We also demonstrate that TDP-43 increases cytosolic Ca^2+^ and decreases mitochondrial Ca^2+^ levels following IP3R-mediated Ca^2+^ release from ER stores, which is consistent with a decrease in ER–mitochondria associations^20^. Our findings that overexpression of wild-type as well as mutant TDP-43 affects ER-mitochondria associations, the VAPB-PTPIP51 interaction and associated Ca^2+^ homeostasis are consistent with many studies which show that wild-type as well as mutant TDP-43 all induce similar, aggressive forms of disease in transgenic mice and in other models (for example, refs 24, 25, 26, 27, 28, 41). Thus, ER–mitochondria and VAPB–PTPIP51 interactions may be targets for damage by TDP-43. The precise mechanisms underlying this damage are not clear. However, in agreement with previous studies we show that overexpression of TDP-43 activates GSK-3β a kinase strongly implicated in both ALS and dementia^30^,^42^,^43^. Moreover, we demonstrate that modulating GSK-3β activity affects the VAPB–PTPIP51 interaction; inhibiting GSK-3β increased, whereas overexpression of GSK-3β decreased binding of VAPB to PTPIP51. Thus, the effect of TDP-43 on the VAPB–PTPIP51 interaction may involve activation of GSK-3β, which then leads to decreased VAPB–PTPIP51 binding. This may be via direct phosphorylation of VAPB or PTPIP51 by GSK-3β to inhibit their binding, or signalling via GSK-3β to downstream effectors that somehow influences VAPB/PTPIP51 phosphorylation and/or binding. Future studies to tackle this will involve mass spectrometric sequencing of VAPB and PTPIP51 to identify any GSK-3β phosphorylation sites, and the analyses of their function using mutagenic approaches.

Disruptions to a number of physiological processes are seen in both ALS and FTD. These include damage to mitochondria and mitochondrial ATP production, disruption to Ca^2+^ homeostasis, perturbation to axonal transport of mitochondria, damage to autophagy and finally damage to the ER including activation of the unfolded protein response (UPR) (see review (ref. 44)). One conundrum is how so many apparently disparate pathological features might link together in a common disease pathway. However, all of these features are regulated by ER–mitochondria associations. Thus ER–mitochondrial Ca^2+^ exchange following release from ER stores affects both cellular Ca^2+^ homeostasis and mitochondrial ATP production since several mitochondrial dehyrogenase enzymes involved in ATP synthesis are Ca^2+^ regulated^4^,^45^. Decreased mitochondrial ATP production is a feature of at least some forms of ALS and FTD^46^. Likewise, ER–mitochondria associations are linked to axonal transport. Mitochondria are anterogradely transported through axons on kinesin-1 motors and attach to kinesin-1 via the outer mitochondrial membrane protein Miro, which acts as a Ca^2+^ sensor; elevated Ca^2+^ levels disrupt transport^47^. Recently, Miro has been shown to localize to ER–mitochondria contact sites and a proportion of ER has been shown to be co-transported with mitochondria^48^,^49^. Also, defects in autophagy are features of ALS/FTD involving TDP-43; autophagosomes are now known to form at ER–mitochondria contacts^7^,^50^,^51^. Finally, ER–mitochondria associations impact on ER stress and the UPR since a number of ER chaperones involved in protein folding localize to MAM and structural uncoupling of ER from mitochondria induces ER stress and ER-UPR^3^,^52^. Indeed, VAPB itself is linked to the UPR (see review (ref. 53)). Thus, the TDP-43 loosening of ER–mitochondria associations that we described here may contribute to some of the pathological features of ALS/FTD.

Several recent studies have implicated defective ER–mitochondria signalling in some related neurodegenerative diseases including Alzheimer’s and Parkinson’s diseases^11^,^12^,^13^,^14^,^15^,^54^. Damage to the ER–mitochondria axis may thus be a common feature of neurodegenerative diseases. The results described here, which show that VAPB and PTPIP51 function to mediate ER–mitochondria associations and which demonstrate that the VAPB–PTPIP51 interaction is disrupted by TDP-43, will facilitate future studies on the role of ER–mitochondria associations in neurodegenerative diseases.

## Methods

### Plasmids and siRNAs

Mammalian expression vectors were HA-tagged PTPIP51 in pCIneo, ECFP-tagged VAPB in pECFP-C1, M3R, EGFP-tagged wild-type TDP-43 and mutants TDP-43Q331K and TDP-43M337V in pEGFP-C1^20^,^55^,^56^. TDP-43A382T and TDP-43G348C mutants were created using a QuikChange XL mutagenesis kit (Stratagene). Mutagenic primers were: TDP-43A382T 5′-GGCTCTAATTCTGGTGCAACAATTGGTTGGGG-3′ and 5′-CCCCAACCAATTGTTGCACCAGAATTAGAGCC-3′; TDP-43G348C 5′-GCAGAACCAGTCATGCCCATCGGGTAATAACC-3′ and 5′-GGTTATTACCCGATGGGCATGACTGGTTCTGC-3′. PTPIP51 was cloned into the internal ribosome entry site vector pIRES2-AcGFP1. GST-VAPB and GST-PTPIP51 fusion plasmids were created by amplifying appropriate VAPB and PTPIP51 sequences by PCR and cloning into pGEX5X (GE Healthcare). Control, VAPB and PTPIP51 siRNAs were from Dharmacon and Origene. Mouse siRNA sequences for NSC34 cells were: VAPB 5′-UGUUACAGCCUUUCGAUUAUU-3′; PTPIP51 5′-GGAUGACAACGCUGGCAAAGGGUCU-3′ and 5′-AGGUUAUACAACAGCCAACGCGGAG-3′; TDP-43 5′-CGAUGAACCCAUUGAAAUA-3′ and 5′-GGAGAGGAUUUGAUCAUUA-3′. Human VAPB and PTPIP51 siRNAs for HEK293 cells were: VAPB 5′-GCUCUUGGCUCUGGUGGUUUU-3′; PTPIP51 5′-CCUUAGACCUUGCUGAGAUUU-3′ (see (ref. 20)).

### Antibodies and inhibitors

Rat and rabbit antibodies to VAPB and PTPIP51 were generated by immunization with GST-VAPB(1–220) and GST-PTPIP51(36–470), respectively^20^. Rabbit anti-PTPIP51 was from Atlas, rabbit anti-haemagglutin (HA), mouse anti-α-tubulin (DM1A) and rabbit anti-Mitofusin-2 were from Sigma. Rabbit anti-TOM20 was from Santa Cruz Biotechnology. Rabbit anti-GFP and mouse anti-βactin were from Abcam. Mouse PDI (RL77) was from Affinity Bioreagents. Rabbit anti-TDP-43 (12892-1-AP) was from Proteintech. Antibodies to total and ser9-phosphorylated (inactive) GSK-3β were from BD Transduction Labs (mouse 610201) and Cell Signalling (rabbit 9336), respectively. The concentrations used for the different antibodies are shown in Supplementary Table 1. Secondary antibodies were horseradish peroxidase-coupled goat anti-mouse, anti-rabbit and anti-rat Igs (GE Healthcare), and Alexa fluorophore (350, 488 and 568)-coupled goat anti-mouse and anti-rabbit IgGs (Invitrogen). AR-A014418 and CT99021 were from Abcam and Cayman, respectively.

### Cell culture and transfection

NSC34 cells were provided by Professor Dame Pamela Shaw, (University of Sheffield, UK). CV1 and HEK293 cells were obtained from the ATCC. Cells were grown in Dulbecco’s modified Eagle’s medium containing 10% fetal bovine serum supplemented with 2 mM glutamine. NSC34 and HEK293 cells were transfected using Lipofectamine 2000 (Invitrogen); CV1 cells were transfected using Exgen (Fermentas) according to the manufacturer’s instructions. Transfected cells were analysed 36 h post transfection; siRNA-treated cells were analysed 4 days post treatment. For fluorescence-activated flow cytometry cell sorting, NSC34 were trypsinized, resuspended in phosphate-buffered saline (PBS) and then sorted for ECFP and EGFP expression using a BD FACSAria cell sorter, re-plated and cultured for a further 16 h before processing.

### Electron and light microscopy

For EM, NSC34 cells were fixed with 2.5% glutaraldehyde in 0.1 M sodium cacodylate buffer (pH 7.2) for 3 h and then harvested by scraping gently with a plastic scraper. The cells were pelleted by centrifugation at 800*g*(av) for 10 min, washed in buffer and post-fixed for 1 h in 1% osmium tetroxide in 0.1 M sodium cacodylate buffer. The cells were then stained for 1 h with 1% uranyl acetate in water before dehydration and embedding in Taab resin. Spinal cords from 4% paraformaldehyde-perfused mice were immersed in 2.5% glutaraldehyde in 0.1 M sodium cacodylate buffer, post-fixed in 1% osmium tetroxide, dehydrated in alcohol and propylene oxide and finally infiltrated and embedded in Epon 812 (ref. 26). Samples were viewed on a Tecnai 12 electron microscope at 6,800 or 23,000 magnification. Digital images were acquired and the circumference of each mitochondria and the proportions of the mitochondrial surface (circumference) closely associated (<30 nm) with ER were calculated. Cells were randomly selected for analyses without prior knowledge of transfected plasmid, siRNA or mouse genotype. All clearly identified mitochondria in the samples were scored. Image analyses were performed using Adobe Illustrator and ImageJ.

For immunostaining of CV1 cells, cells were fixed in 4% paraformaldehyde/0.1% glutaraldehyde in PBS, quenched in 0.05 M ammonium chloride in PBS and permeabilized with 0.25% Triton X-100 in PBS. Following blocking in 3% bovine serum albumin in PBS, samples were incubated with primary antibodies diluted in blocking solution, washed with PBS and incubated with secondary antibodies. Following washing, the samples were mounted in Mowiol-DABCO mounting medium containing 10% (w/v) Mowiol 4–88 (Calbiochem), 25% (w/v) glycerol and 2.5% (w/v) DABCO (1,4-diazobicyclo[2.2.2]octane) in 100 mM Tris–HCl pH 8.5. Cells were imaged using a Zeiss LSM510Meta confocal microscope equipped with a × 63 Plan-Apochromat 1.4 NA objective and analysed using ImageJ with the ICA plug-in^57^. Statistical analyses were performed using GraphPad Prism.

### SDS-PAGE, immunoblotting, immunoprecipitation and GST assays

Cells were harvested for SDS-PAGE and immunoblotting by scraping into SDS–PAGE sample buffer containing 2% SDS, 100 mM dithiothreitol, 10% glycerol, 0.1% bromophenol blue and protease inhibitors (Complete Roche) in 50 mM Tris–HCl pH 6.8 and heating to 100 °C for 5 min^57^. For immunoprecipitation assays, transfected HEK293 cells were lysed in ice-cold immunoprecipitation buffer comprising 50 mM Tris-citrate pH 7.4, 150 mM NaCl, 1% Triton X-100, 5 mM ethylene glycol tetraacetic acid, 5 mM EDTA and protease inhibitors (Complete, Roche) for 30 min. Transgenic mouse samples were homogenized and diluted into the same buffer. NSC34 cells were lysed in RIPA buffer containing 25 mM Tris–HCl pH 7.5, 1% NP-40, 1% sodium deoxycholate, 0.1% SDS, 150 mM NaCl and protease inhibitors (Complete Roche). The samples were then centrifuged at 13,000*g* for 20 min and the supernatants were transferred to fresh tubes and precleared by incubation with Protein-G-sepharose beads (prepared as a 50% slurry in PBS containing 0.1% Triton X-100). Following centrifugation at 2,000 *g* for 30 s to settle the beads, supernatants were transferred to fresh tubes and protein concentrations determined using a Bio-Rad protein assay kit. The protein concentrations were adjusted to 1 μg μl^−1^, and 500 μg protein incubated with appropriate primary antibodies on a rotary shaker for 16 h at 4 °C. Antibodies were then captured by addition of 30 μl Protein-G-sepharose beads (50% slurry in PBS/0.1% Triton X-100). Following washing in PBS/0.1% Triton X-100, bound proteins were prepared for SDS–PAGE by addition of 50 μl SDS-PAGE sample buffer and heating to 100 °C. Signals on immunoblots were quantified using ImageJ after scanning with an Epson Precision V700 Photo scanner essentially as described by us in previous studies^57^. To ensure the signals obtained were within the linear range, the mean background-corrected optical density (OD) of each signal was interpolated for an OD calibration curve created using a calibrated OD step tablet (Kodak). Only film exposures that gave OD signals within the linear range of the OD calibration curve were used for the statistical analyses.

GST, GST-VAPB and GST-PTPIP51 fusion proteins were prepared on glutathione-sepharose beads from *E. coli* BL21(DE3) according to the manufacturer’s instructions (GE Healthcare). For cellular pull-down assays, GST and GST fusion proteins were incubated with transfected cell lysates prepared in immunoprecipitation buffer as described above^58^. For *in vitro* pull-down assays of recombinant VAPB and PTPIP51, the cytoplasmic domain of PTPIP51 (aa36–470), was prepared as a GST fusion protein and PTPIP51(36–470) released from the GST moiety by incubating GST-PTPIP51(36–470) bound to glutathione-sepharose beads with 1 μg factor XA protease in 50 μl buffer containing 20 mM Tris–HCl pH 8.0, 100 mM NaCl, 2 mM CaCl_2_ for 16 h at 4 °C according to the manufacturer’s recommendations (New England Biolabs). 50 μg of purified recombinant PTPIP51(36-470) was then incubated for 16 h at 4 °C with either GST or GST-VAPB(1–220) fusion proteins on glutathione-sepharose beads in PBS containing 1% Triton X-100. GST-fusion protein complexes were pelleted and washed three times with incubation buffer by centrifugation at 2,000 *g* for 30 s, and analysed by SDS-PAGE and immunoblotting as described above.

Full blots are shown in Supplementary Fig. 1.

### Ca^2+^ measurements

Cytosolic and mitochondrial Ca^2+^ levels were measured following IP3R-mediated release from ER stores in HEK293 cells^20^. To do so, cells transfected with M3R and either empty vector control plasmid or TDP-43 plasmids were loaded with either 2 μM Fluo4-AM or Rhod2-AM dye (Invitrogen) in external solution (145 mM NaCl, 2 mM KCl, 5 mM NaHCO_3_, 1 mM MgCl_2_, 2.5 mM CaCl_2_, 10 mM glucose and 10 mM Na-HEPES pH 7.25) containing 0.02% Pluronic-F27 (Invitrogen) for 15 min at 37 °C, followed by washing in external solution for 15 min. Fluo4 and Rhod2 fluorescence were timelapse recorded (1-s intervals) with MetaMorph (Molecular Dynamics) on an Axiovert S100 microscope (Zeiss) equipped with appropriate filtersets (Chroma Technology), a × 40/1.3NA Plan-Neofluar objective (Zeiss) and a Photometrics Cascade-II 512B EMCCD. The cells were kept under constant perfusion with external solution (0.5 ml min^−1^). IP3R-mediated Ca^2+^ release from ER stores was triggered by application of 100 μM Oxotremorine-M (Tocris) for 2 min. Ca^2+^ levels were calculated as relative Fluo4 or Rhod2 fluorescence compared with baseline fluorescence at the start of the measurement.

## Additional information

**How to cite this article:** Stoica, R. *et al*. ER–mitochondria associations are regulated by the VAPB–PTPIP51 interaction and are disrupted by ALS/FTD-associated TDP-43. *Nat. Commun.* 5:3996 doi: 10.1038/ncomms4996 (2014).

## Supplementary Material

Supplementary Information Supplementary Figure 1 and Supplementary Table 1
