# Supplementary material for: ER–mitochondria associations are regulated by the VAPB–PTPIP51 interaction and are disrupted by ALS/FTD-associated TDP-43
Source: Nat Commun. 2014 Jun 3;5:3996. doi: 10.1038/ncomms4996 (PMC4046113; doi:10.1038/ncomms4996)
Supplement: Supplementary Information — Supplementary Figure 1 and Supplementary Table 1 [file ncomms4996-s1.pdf]

Supplementary Figure 1. Full immunoblots with indicated areas of selection

Figure 1A

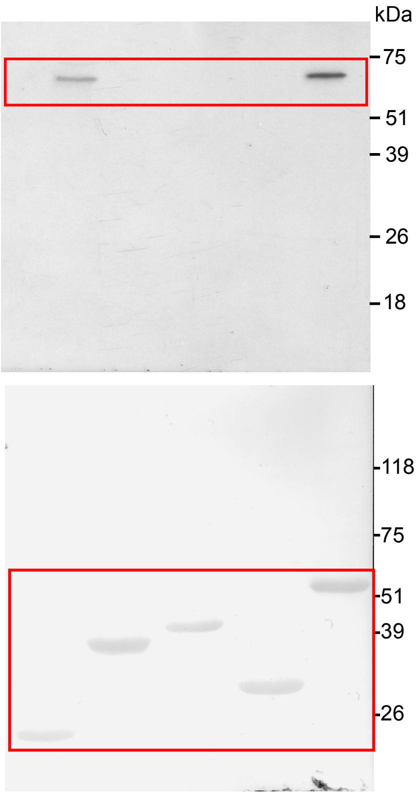


Figure 1B

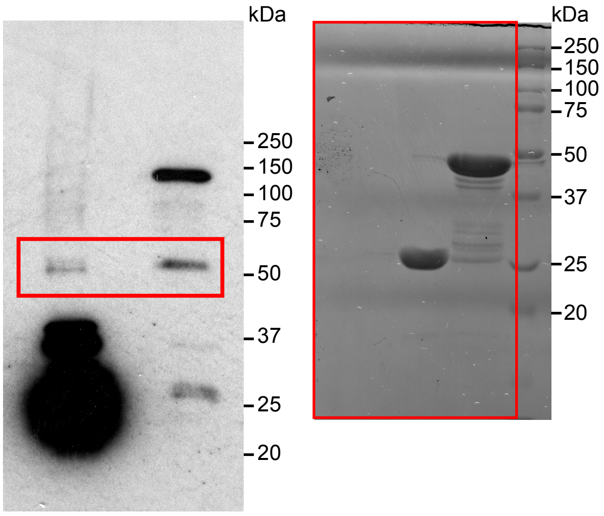


Figure 2A

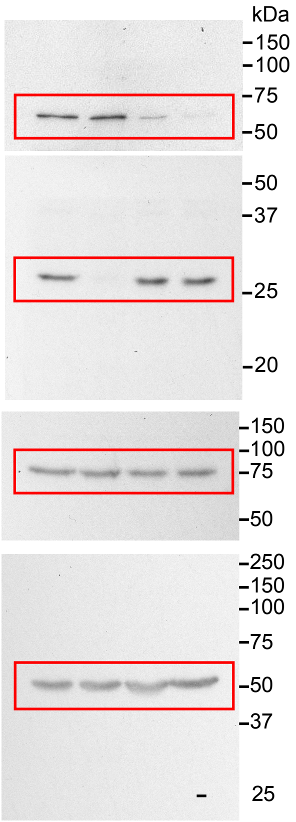


Figure 5B

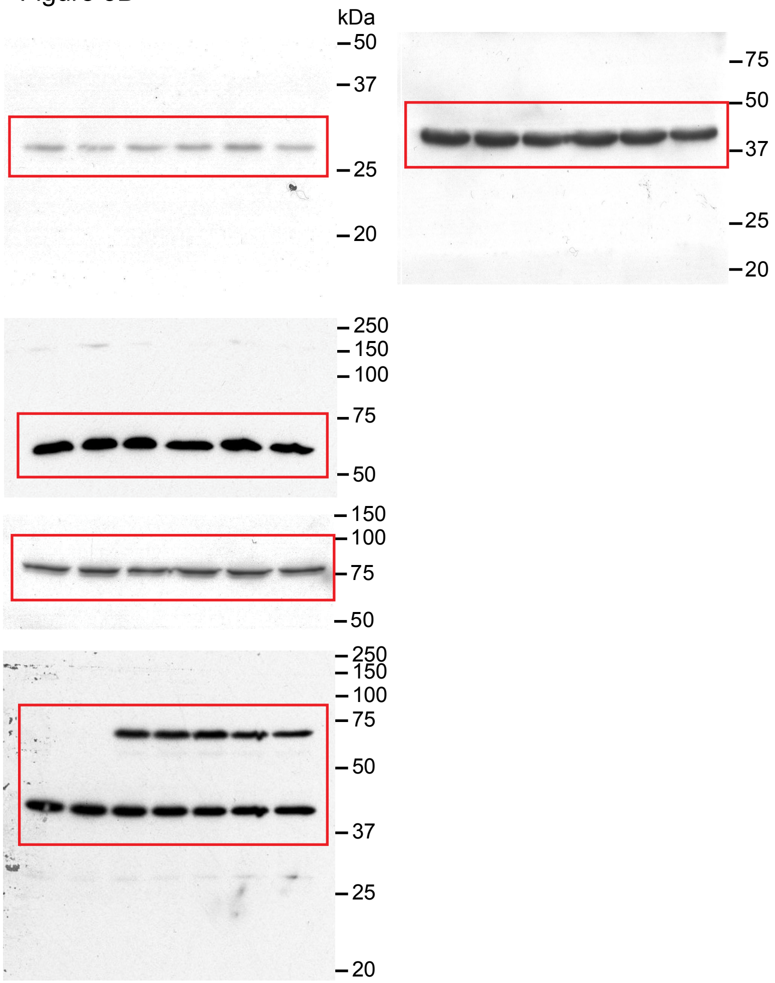


Figure 6A

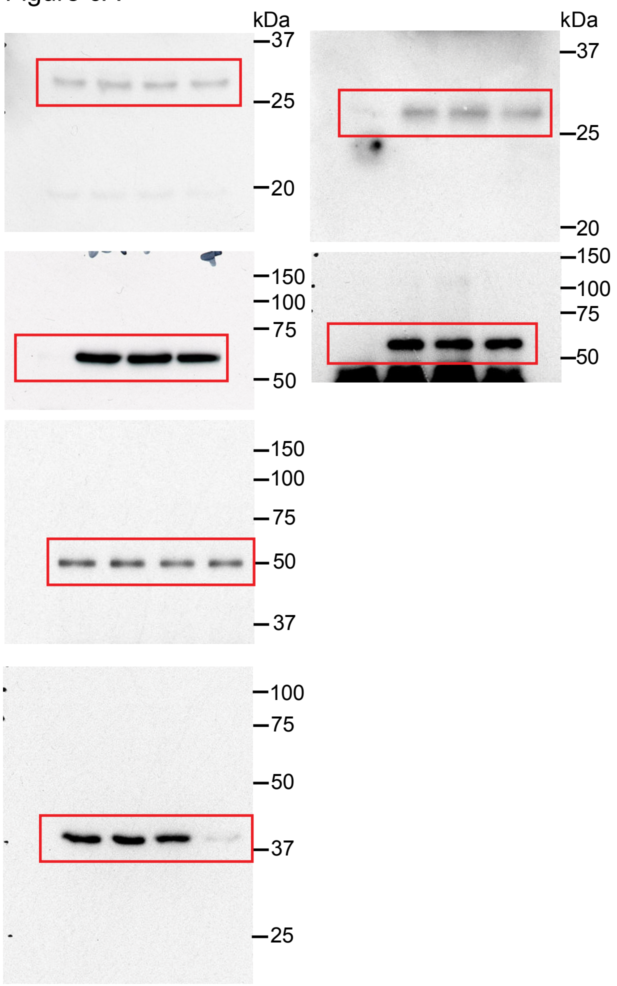

Supplementary Figure 1. Continued

Figure 7A

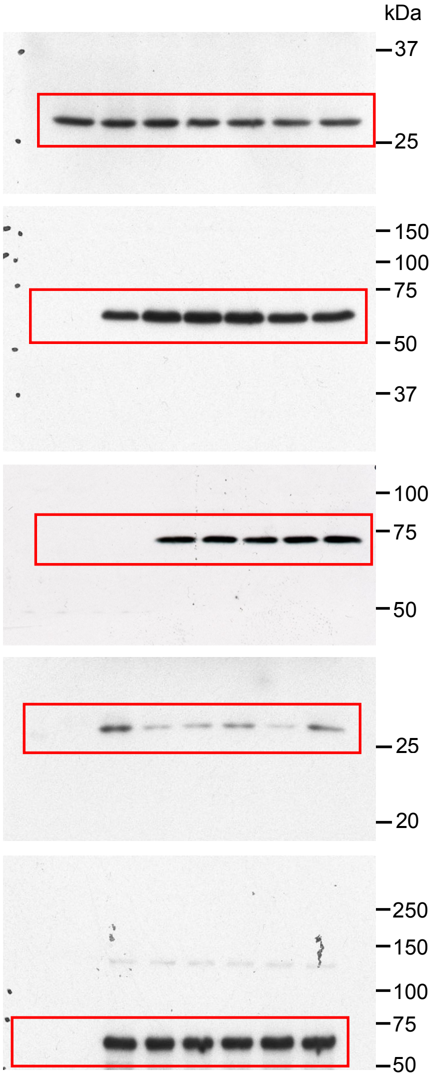


Figure 7B

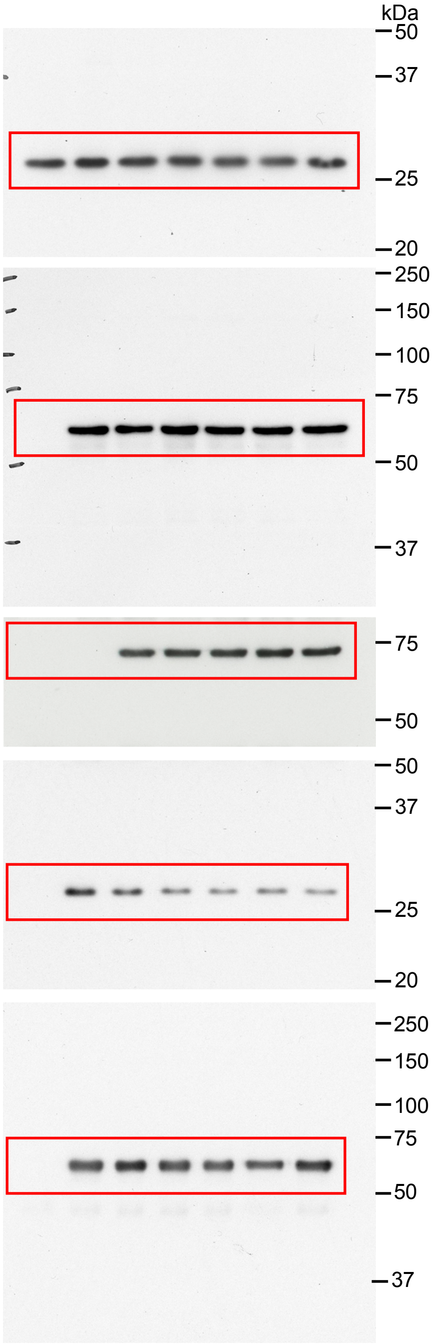


Figure 7D

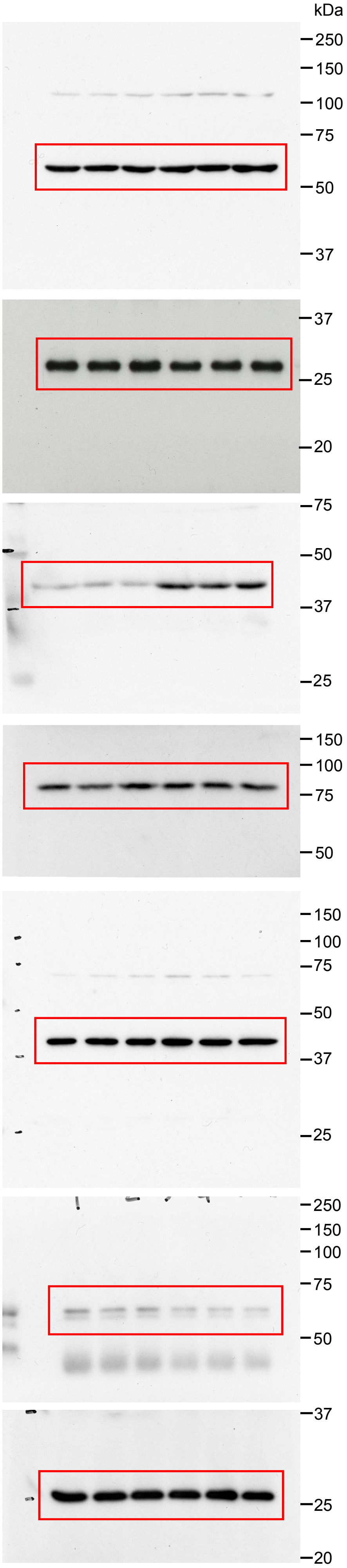

Supplementary Figure 1. Continued

Figure 9A

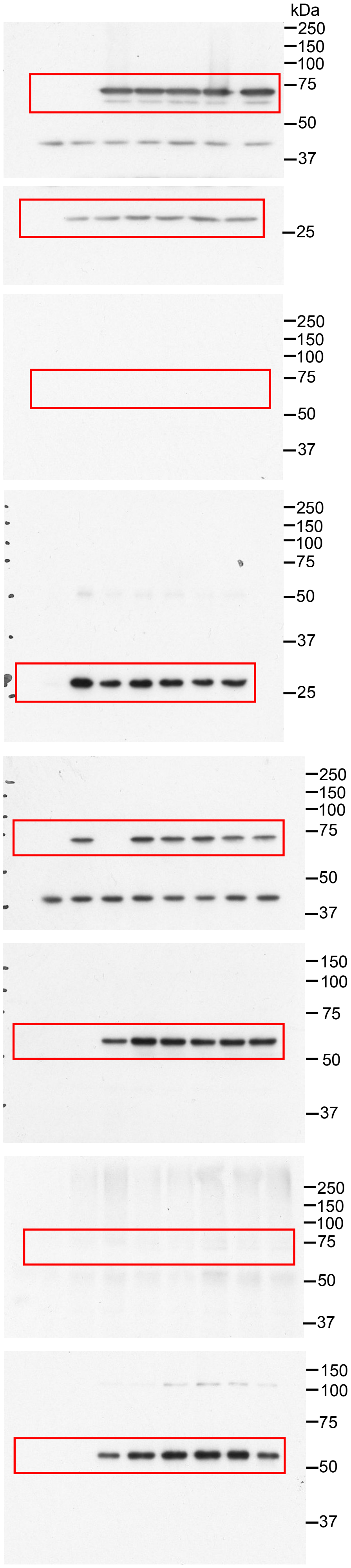


Figure 9B

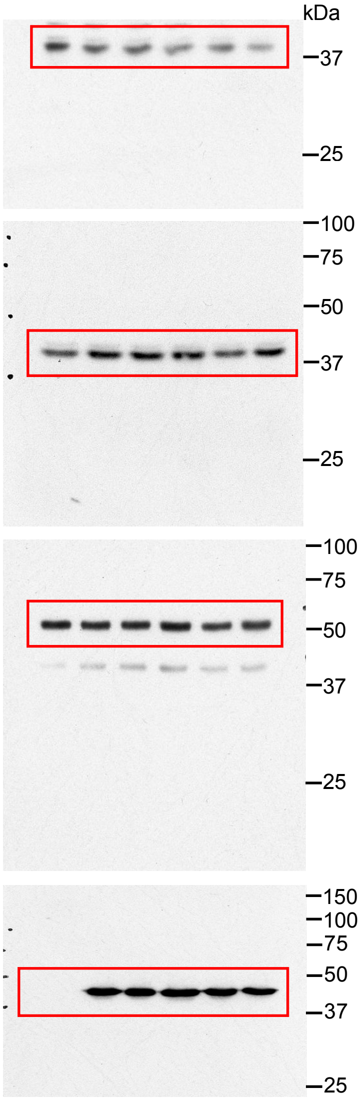


Figure 9C

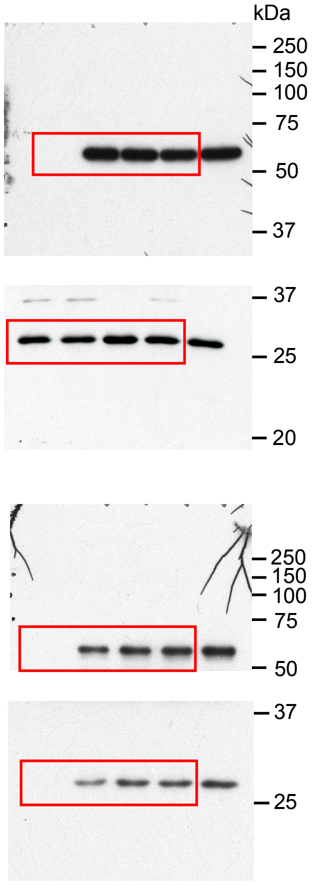


Figure 9D

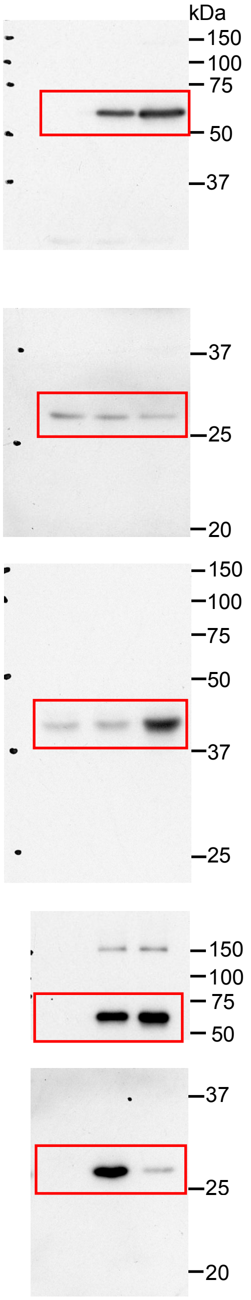

Supplementary Table 1. Primary antibody concentrations

| Antibody | Supplier | Species | Working dilution |
|---|---|---|---|
| VAPB (#SK83) | Reference 20 | Rat | 1:2000 (IB) |
| VAPB (#3503) | Reference 20 | Rabbit | 1:200 (IP) |
| PTPIP51 (#skr10) | Reference 20 | Rat | 1:1000 (IB) |
| FAM82A2 (PTPIP51) | Atlas Antibodies | Rabbit | 1:1000 (IB) |
| HA | Sigma | Rabbit | 1:5000 (IB)<br>1:300 (IP) |
| α-tubulin (DM1A) | Sigma | Mouse | 1:40,000 (IB) |
| GFP | Abcam | Rabbit | 1:5000 (IB) |
| Actin (AC-15) | Abcam | Mouse | 1:15,000 (IB) |
| PDI (RL77) | Affinity BioReagents | Mouse | 1:200 (IF) |
| TDP-43 | Proteintech | Rabbit | 1:5000 (IB) |
| 9B11 (myc-tag) | Cell Signaling | Mouse | 1:5000 (IB)<br>1:300 (IP) |
| TOM20 | Santa Cruz Biotechnology | Rabbit | 1:200 (IF) |
| Mitofusin2 | Sigma | Rabbit | 1:5000 (IB) |
| GSK3β | BD Transduction Laboratories | Mouse | 1:2000 (IB) |
| GSK3β-S9 | Cell Signalling | Rabbit | 1:2000 (IB) |

IB-immunoblot, IF-immunofluorescence, IP-immunoprecipitation
